# Supplementary material for: Clinical outcomes of a community clinic-based lifestyle change program for prevention and management of metabolic syndrome: Results of the ‘Vida Sana/Healthy Life’ program
Source: PLoS One. 2021 Apr 1;16(4):e0248473. doi: 10.1371/journal.pone.0248473 (PMC8016241; doi:10.1371/journal.pone.0248473)
Supplement: Attachment — Submitted filename: Vida Sana Main Effects Response to Reviewers.docx [file pone.0248473.s001.docx]

Response to Reviewers Table

| Reviewer Comment | Revision Citation |
| --- | --- |
| 1. Please ensure that your manuscript meets PLOS ONE's style requirements, including those for file naming. The PLOS ONE style templates can be found at  <https://journals.plos.org/plosone/s/file?id=wjVg/PLOSOne_formatting_sample_main_body.pdf> and  <https://journals.plos.org/plosone/s/file?id=ba62/PLOSOne_formatting_sample_title_authors_affiliations.pdf> |  |
| 2. In your Methods section, please provide additional information regarding the program. If materials, methods, and protocols are well established, authors may cite articles where those protocols are described in detail, but the submission should include sufficient information to be understood independent of these references (<https://journals.plos.org/plosone/s/submission-guidelines#loc-materials-and-methods>); for example, the role of "navegantes" needs to be explained in more detail. | We have a protocol paper that details all of this information. We are trying to balance the need to provide information to readers of this paper with not repeating what is already published.  Risica PM, McCarthy M, Barry K, Oliverio SP, De Groot AS. Community clinic-based lifestyle change for prevention of metabolic syndrome: Rationale, design and methods of the 'Vida Sana/healthy life' program. Contemp Clin Trials Commun. 2018 Oct 25;12:123-128. doi: 10.1016/j.conctc.2018.10.002. PMID: 30417157; PMCID: PMC6218840.  Additional details regarding the role of Navegantes have been added (page 6 line 102)  “*Navegantes* are available during all clinical encounters CEHC, serving as liaisons for most patients between patients and the healthcare system by offering support relating to insurance options, specialty referrals, and social determinants of health. They are specifically trained to provide nutritional and chronic disease information and advice, which may include offering enrollment into the Vida Sana Program and can act as advocates for the individual patients navigating the healthcare system and the clinic. ” |
| Moreover, please ensure you have provided sufficient details to replicate the analyses such as:  a) the recruitment date range (month and year), | This has been added (January 2014 - June 2017) on page 5, lines 94-95. |
| b) a description of any inclusion/exclusion criteria that were applied to participant recruitment, | We appreciate the reviewer’s helpful assessment and this specific suggestion for the paper. Information regarding recruitment is found on page 6, line 114. Inclusion and exclusion criteria are not highly specific, as VSP is a program, not following a tight research protocol. |
| c) a table of relevant demographic details, | Thank you for the suggestion and we have included this information in Table 1. |
| d) a statement as to whether your sample can be considered representative of a larger population, | Thank you for the suggestion. We compared the clinical risk of the VSP study participants with other studies (pages 19-20, lines 274-283). Also, we addressed our concerns about potential generalizability to other clinical settings on page 23, line 343. |
| e) a description of how participants were recruited | Thank you for the suggestion and we have included this information on Page 6, line 114. |
| f) descriptions of where participants were recruited and where the research took place. | Thank you for the suggestion and we have included this information on Page 6, line 114. |
| 3. We noticed you have some minor occurrence of overlapping text with the following previous publication(s), which needs to be addressed:  - <https://www.sciencedirect.com/science/article/pii/S2451865418301005?via%3Dihub>  The text that needs to be addressed involves some sentences of the Introduction.  In your revision ensure you cite all your sources (including your own works), and quote or rephrase any duplicated text outside the methods section. Further consideration is dependent on these concerns being addressed. | Thank you for pointing this out. The overlapping text has been identified and paraphrased, with appropriate references included. See revised text on pages 4-5, lines 69-45. |
| 4. We note that you have included the phrase “data not shown” in your manuscript. Unfortunately, this does not meet our data sharing requirements. PLOS does not permit references to inaccessible data. We require that authors provide all relevant data within the paper, Supporting Information files, or in an acceptable, public repository. Please add a citation to support this phrase or upload the data that corresponds with these findings to a stable repository (such as Figshare or Dryad) and provide and URLs, DOIs, or accession numbers that may be used to access these data. Or, if the data are not a core part of the research being presented in your study, we ask that you remove the phrase that refers to these data. | When “data not shown” was written in the text, it was intended to mean that results of certain analyses were not included in the paper due to limits on how many tables we could include in the paper. |
| 5. We note that you have indicated that data from this study are available upon request. PLOS only allows data to be available upon request if there are legal or ethical restrictions on sharing data publicly. For more information on unacceptable data access restrictions, please see <http://journals.plos.org/plosone/s/data-availability#loc-unacceptable-data-access-restrictions>. | As per our cover letter, we have chosen to not make the data publicly available. This data is from a small, vulnerable population, such that the information provided in this dataset would potentially be identifiable due to very small numbers in some demographic groups. We are happy to provide the data to any researcher or other professional that would like access to it. No reasonable request for the data will be refused. Dr. Annie De Groot, Medical Director of Clinica Esperanza/Hope Clinic senior author on this paper is the right person to contact for further discussion of the data. |
| In your revised cover letter, please address the following prompts:  a) If there are ethical or legal restrictions on sharing a de-identified data set, please explain them in detail (e.g., data contain potentially sensitive information, data are owned by a third-party organization, etc.) and who has imposed them (e.g., an ethics committee). Please also provide contact information for a data access committee, ethics committee, or other institutional body to which data requests may be sent.  b) If there are no restrictions, please upload the minimal anonymized data set necessary to replicate your study findings as either Supporting Information files or to a stable, public repository and provide us with the relevant URLs, DOIs, or accession numbers. For a list of acceptable repositories, please see <http://journals.plos.org/plosone/s/data-availability#loc-recommended-repositories>. | See above |
| Introduction  Minor comment – pg 5, line 88, If this is data is from years 2-5 data, why is the duration of follow-up so short? Would have loved to see the persistence of these changes since it seems like it is just a 12 week intervention and then it is over. Is that the case? Is there not demand from the group members to continue these meetings after | Thank you for bringing this point to our attention. In part, follow-up for VSP is limited due to the transient nature of this population. Even if the patients do stay in the area, they change homes, phones, etc frequently. The only way to keep in touch with them after about 6 months is to see them when they show up or work extremely hard to track them down (through other contacts that they may have provided). Unfortunately, the financial cost sets limitations on staff time and our preference is to focus on offering the program to as many of the thousands of clinic patients as possible. Focusing on long term follow up with all past patients would impede the Navegantes’ ability to offer the same level of education to new patients. CEHC patients also commonly hold multiple jobs and face child care and transportation challenges, so it is hard for them to commit to programs with more sessions. However, offering follow-up sessions with “refreshers” and health checks is something we should consider in the future.  All of the data is collected specifically for this study, not by chart abstraction. |
| Methods  Minor comment – Can you briefly mention how the Navegantes and the study activities integrate with the rest of the clinical team? | Thank you for pointing this out, as the integration of the Navegantes and Vida Sana is important to the structure of our clinic. The following text is added on page 6, line 107.:  “*Navegantes* are available during all clinical encounters CEHC, serving as liaisons for most patients between patients and the healthcare system by offering support relating to insurance options, specialty referrals, and social determinants of health. They are specifically trained to provide nutritional and chronic disease information and advice, which may include offering enrollment into the Vida Sana Program and can act as advocates for the individual patients navigating the healthcare system and the clinic.” |
| Minor comment – pg 7 , line 138, are there any guidelines which suggest that a fasting blood sugar with four hours of fasting and using a point of care glucose meter is adequate for monitoring individual patient care? If not, please highlight this as a limitation as I can understand your reasons for using a point of care test. It is worth noting that there are accuracy concerns with point of care meters. | This is an important point, as there are certainly limitations to using point-of-care glucose testing. Previous studies have shown that values do not differ significantly between fingerstick point-of-care and serum laboratory testing. However, we should note that a single blood glucose test is not necessarily representative of a patient’s overall clinical picture, and A1c values should be considered more. We have added this reference to address the point.  Lacara T, Domagtoy C, Lickliter D, Quattrocchi K, Snipes L, Kuszaj J, Prasnikar M. Comparison of point-of-care and laboratory glucose analysis in critically ill patients. Am J Crit Care. 2007 Jul;16(4):336-46; quiz 347. PMID: 17595363. |
| Minor comment – pg 9, line 171, Slightly confused about the imputation of missing values. Did you do a calculation to predict missing data or did you just carry the last value forward. I think your wording is clear but just wanted to confirm. My one concern with this is that with >30% lack of completion, this might really impact the analyses | The last value was carried forward for each patient that did not complete the program.  Our retention rate is similar to other community studies, which is described beginning on page 22. Line 343. |
| Minor comment – Do you have any comment on the sample size considerations for this intervention? What clinically significant differences in outcomes were you designing your study to assess? It seems like there were many significant differences noted but some of minimal clinical significance. | Thank you for this comment. This paper is not describing a study, so we did not calculate an a priori planned sample size before the study. Instead, we conducted post-hoc analysis from participants. We have added a statement to this effect beginning on page 6, line 122. |
| Stylistic comment - I would have like to also see some kind of composite outcome if you could imagine one for this work. I can’t think of one outside of maybe the AHA CV risk score but would be a nice thing to add for future studies. | We thank the reviewers for this excellent idea, and we will be updating our data collection moving forward so that future analyses can use this ASCVD score. However, we do not have smoking status, HTN treatment status, or HDL cholesterol. The score can also not be used for patients under 40, which would limit our analysis.  <http://tools.acc.org/ASCVD-Risk-Estimator-Plus/#!/calculate/estimate/>  <https://www.mdcalc.com/framingham-risk-score-hard-coronary-heart-disease> |
| Results  Minor comment - Pg 12 second paragraph. Can you comment further in the discussion about why people with higher a1c or glucose were less likely to complete the intervention? Do you have any sense of the number of other appointments they had which may explain why they skipped this appointment? | This is another good question about the other appointments they may have not attended, and overall, “compliance,” so to speak, with clinical care. We do not have that information available, but it will be considered moving forward. We anecdotally hear from patients and might suggest that those with worse diabetes metrics may have been those who faced larger structural barriers to getting medical care, such as lack of a stable living situation, difficulty with transportation, child care or work schedules. This is noted beginning on page 22, line 350.  Additionally, this is why we do ITT analyses. We anticipate that participants who are not retained are more likely to have less-positive changes, which we account for by assuming that they have no improvement from baseline. |
| Minor comment – page 13, table 2 – what is in the parentheses for knowledge assessment? | See methods section where the knowledge assessment is described (page 9, line 176). |
| Discussion  Stylistic comment - can you talk about the costs of this program? | We agree that costs for a program such as this will be important to readers. We have added a section addressing costs beginning on page 24, line 376. |
| Minor Comment - Past studies have often shown that men tend to have less uptake in these kinds of groups. Can you comment on that in the discussion or any adaptations you would suggest going forward. | We agree that this is a concern for this and other studies. We have added a comment and reference regarding participation of men in VSP and other previous studies beginning on page 22, line 352. |
| Major comment - Pg 18, 4th paragraph – There is a significant typo – “reduction of 8.3 mg/dL in A1c was observed”. I’m assuming this should be fasting blood sugar instead of A1c. | Thank you for picking up this mistake. Yes, it should be glucose. We have corrected the MS. Thank you very much. |
| **Comments to the Author**  1. Is the manuscript technically sound, and do the data support the conclusions?  The manuscript must describe a technically sound piece of scientific research with data that supports the conclusions. Experiments must have been conducted rigorously, with appropriate controls, replication, and sample sizes. The conclusions must be drawn appropriately based on the data presented.  Reviewer #1: Partly  2. Has the statistical analysis been performed appropriately and rigorously?  Reviewer #1: Yes  3. Have the authors made all data underlying the findings in their manuscript fully available?  The [PLOS Data policy](http://www.plosone.org/static/policies.action#sharing) requires authors to make all data underlying the findings described in their manuscript fully available without restriction, with rare exception (please refer to the Data Availability Statement in the manuscript PDF file). The data should be provided as part of the manuscript or its supporting information, or deposited to a public repository. For example, in addition to summary statistics, the data points behind means, medians and variance measures should be available. If there are restrictions on publicly sharing data—e.g. participant privacy or use of data from a third party—those must be specified.  Reviewer #1: No  4. Is the manuscript presented in an intelligible fashion and written in standard English?  PLOS ONE does not copyedit accepted manuscripts, so the language in submitted articles must be clear, correct, and unambiguous. Any typographical or grammatical errors should be corrected at revision, so please note any specific errors here.  Reviewer #1: Yes  5. Review Comments to the Author  Please use the space provided to explain your answers to the questions above. You may also include additional comments for the author, including concerns about dual publication, research ethics, or publication ethics | We appreciate the reviewer’s assessment. This study was not a randomized trial as the clinic does not have the resources and personnel to do that.  Regarding the data availability, we will make deidentified data available upon request. |
| Reviewer #1: General Comments  Overall this is a very well written paper on an interesting topic – a community clinic-based lifestyle change program for an underserved, largerly Spanish-speaking community in Rhode Island. The authors should be commended for their very clear description of the methods and the results sections in particular. This manuscript would certainly add to the literature. However, I do have some concerns that the authors’ conclusions do not reflect their findings. In particular, although the changes in glucose, cholesterol and blood pressure were statistically significant for all participants, they do not seem clinically impressive.  Additionally, the baseline knowledge of participants seemed quite high, and it is likely that systemic problems such as access to inexpensive healthy foods, increased stress levels, and limited time available greatly impact disease management and diet/exercise modification. It would be great to see the authors incorporate some discussion about the fact that these types of community interventions unfortunately are unable to tackle the larger structural inequities at play and that relatively high baseline knowledge levels among participants potentially indicates that literacy is likely only one small factor that needs to be addressed.  Other Comments  1) Title: The title implies that this program is for ‘prevention’ of metabolic syndrome. However, it seems that some participants already had metabolic syndrome at baseline. Please clarify.  2) Lines 74-77: Recommend adding a brief explanation of why these behavioral risk factors are higher in Hispanics compared to non-Hispanic whites – i.e. systems of oppression/structural inequities upstream likely leading to racial/ethnic behavioral differences. Might be interesting to add additional evidence from the Healthy Americas Survey such as question HA-13 regarding cost of fresh fruits and vegetables, how do whites vs Hispanics respond to this question?  3) Line 88: please include the years during which the study took place since readers will not know when years 2-5 were.  4) Lines 103-104: Since recruitment extends to community members interested in the program, I do wonder if this incorporates a selection bias? Did the authors compare outcomes between those who opted into the program to others?  5) Lines 107-111: Would be helpful if the authors could comment in the discussion on how they envision this program being sustainable given that it seems to cost >$30/participants not including any cost of the materials, on-site child care services, and Navegantes. I am curious if any of the participants mentioned using the money to buy healthy foods? If so, this should be mentioned as a potential driver of behavior change as well.  6) Line 118: please correct reference 10 as it is written ‘in submission’ and update the reference appropriately.  7) Lines 138-143: For clinical purposes, fasting glucose and cholesterol are usually measured after 8 hours of fasting. Therefore, the results in this study may not correspond to true clinical fasting glucose or cholesterol measurements and should be mentioned as a limitation or clarified if a different guideline was used.  8) Line 164: It would be helpful to understand why SBP of 130 and DBP of 80 were selected as the high-risk blood pressure cut off and provide a reference for the guideline used.  9) Line 169: Given that a parametric test was utilized, please comment regarding the distribution of the population. If the population was not normally distributed, were corresponding non-parametric statistical analyses performed for comparison to ensure consistency in statistical significance?  10) Table 1: This is an excellent table. It is interesting that it seems participants who did not complete the program were more likely to have uncontrolled diabetes with A1Cs>7.0. I would be interested to know if these participants were more likely to be uninsured or if they had other socioeconomic constraints that both impacted their ability to manage their DM as well as attend classes. The authors could consider expanding on this in the discussion if this data is available.  11) Table 3: This is also a great table. However, although most of these variables are statistically significant, they do not seem clinically impressive, especially in the all participants group. I think that this point needs to come across in the discussion and needs to be strongly considered in terms of whether or not this program is truly impacting all participants’ metabolic syndrome. For high risk participants, the A1C decrease of 0.39 in a two month period as well as the systolic BP drop by 10mmHg should be highlighted in the discussion and perhaps the authors might consider that this program should target participants who are highest risk in the future.  12) Paragraph 7 discussion, “Changes in indicators of diabetes….”: In the sentence, Among those beginning VSP with elevated blood glucose levels, a reduction of….in ‘A1C’ I believe should be ‘glucose’.  13) Overall in the discussion, it is unclear how much information was known about participants’ chronic disease management and if many of them were already on or potentially started on medications during the study. It would be helpful if the authors could include more information about this in the methods since participants who opt to join this type of program may also be opting to attend their clinical visits and participate in medication management.  14) Regarding lipid levels, it would be helpful to understand why the authors chose cholesterol levels rather than calculating ASCVD risk for each participant as it seems like the information needed to calculate this score was available.  15) The paragraph about strengths is excellent. If the authors have any additional qualitative data that were collected about the social connection and individual connection with Navegantes, it would be worthwhile to add this formally to the results. If this program is ongoing, it would be very interesting to conduct a qualitative study with participants and Navegantes to further explore this social aspect and the impact this played in suspected behavioral change.  16) The sentence, “The reach of well over 600 participants for this small….indicating the need and potentially very strong benefit for VSP” is strongly worded. This study does demonstrate a significant increase in several vital signs and lab variables, however, as the authors mention, the change may very well have been associated with medications or other factors that were not accounted for. Would recommend altering this to reflect results. | The findings presented in this paper of changes found with participation in VSP are compared to other clinical studies. We examined the number of participants meeting the standard of 5% weight loss (see page 14), which is an indicator of, “clinical significance.”  For other values (chemistries), “clinically significant,” parameters are not established.  However, we agree with the reviewer that the clinical changes found are not dramatic. We have revised our wording to mention that this is only an 8 week study, so a large, clinically significant change might not be expected.  Also, it is important to consider that we are examining the mean changes for a population, not individual clinical changes.  We are grateful to the reviewer for calling attention to the structural inequities: The patients at our clinic and in the Vida Sana program are certainly facing numerous structural inequities that negatively impact their health and access to healthcare. This is noted in the discussion (page 223, beginning on line 355).  Additionally, we have added a more detailed description of the CEHC patient population, which might better describe the challenges mentioned here beginning on page 25, line 409.  1) We agree, and have revised the title to indicate both prevention and management of metabolic syndrome.  2) The introduction has been revised to better describe these behavioral risk factors (see pages 4-5, lines 69-85) Please note that we have included (and are retaining) data from the Healthy Americans Survey, which is reference #9.  3) Patients included in this analysis began the program between January 2014 and June 2017.  4) Method of enrollment into the program is not currently tracked, so we did not assess the differences between these groups. We agree that this would be interesting to be able to assess in the future differences between CEHC and external participants regarding demographics, risk factors and motivation, as well as outcomes.  We also acknowledge that this study, healthy volunteer bias, which is common in most, if not all health improvement intervention studies.  DiMartino LD, Hammill BG, Curtis LH, Gottdiener JS, Manolio TA, Powe NR, Schulman KA. External validity of the cardiovascular health study: a comparison with the Medicare population. Med Care. 2009 Aug;47(8):916-23. doi: 10.1097/MLR.0b013e318197b104. PMID: 19597373; PMCID: PMC2716425.  5) The grocery gift cards are given along with recipes using healthy foods after cooking demonstrations in the class, so our hope is that participants use the money to replicate the new nutritious meals that they learned.  Are actively seeking outside investment in this program including collaboration with other programs (e.g. WISEWOMAN) to provide what might be otherwise unaffordable. We have expanded our comments on cost beginning on page 24, line 375.  6) This reference has been revised.  7) Thank you for pointing out this limitation, which should be mentioned. Since our sessions often occurred in the evenings, it was not feasible to ask all patients to strictly fast for 8 hours, so some values may not be entirely accurate. We have addressed this limitation on page 23, starting on line 370.  8) Updated guidelines from the American Heart Association (and others) classify BP >130/80 as stage 1 hypertension. We have added a reference for this and all cut points defined in these analyses beginning on page 9, line 185.  9) We appreciate this concern. All variables were assessed for normality of distribution and found to be acceptable.  10) We agree and answered this concern shared by Reviewer 1 above.  11) We agree and answered this concern shared by Reviewer 1 above.  12) Yes, we agree, and have fixed this.  13) We agree with the reviewer that this is an important point. However, this data was not collected for this study. We will certainly consider it for future studies.  14) This is a great idea, and we should consider updating our data collection moving forward so that future analyses can use this ASCVD score. However, we do not have smoking status, HTN treatment status, or HDL cholesterol. The score can also not be used for patients under 40, which would limit our analysis.  <http://tools.acc.org/ASCVD-Risk-Estimator-Plus/#!/calculate/estimate/>  15) We completely agree, but have not yet had the resources to conduct this research. Future data collection to assess the social connection between Navegantes and the community is an excellent idea.  16) This sentence will be updated to reflect the other factors that could have affected results. See page 22 beginning on line 322. |
